# Impact of COVID-19 Pandemic on Seroprevalence of HIV, HBV, HCV and HTLV I-II in a Blood Bank in Medellín, Colombia, 2019–2022

**DOI:** 10.3390/tropicalmed8020118

**Published:** 2023-02-14

**Authors:** Jaiberth Antonio Cardona-Arias, Luis Felipe Higuita-Gutiérrez

**Affiliations:** 1 Escuela de Microbiología, Universidad de Antioquia, Calle 70 No. 52–21, Medellín 050012, Colombia; 2 Facultad de Medicina, Universidad Cooperativa de Colombia, Medellín 050012, Colombia

**Keywords:** COVID-19, HIV, HBV, HCV, HTLV I-II, epidemiological surveillance

## Abstract

The objective of this research was to analyze the impact of the COVID-19 pandemic on seroprevalence of HIV, HBV, HCV and HTLV I-II in donors from a blood bank in Medellin, Colombia, 2019–2022. A cross-sectional analytical study was carried out with three groups: pre-pandemic with 14,879 donors; preventive isolation with 9035; and selective isolation + new normality with 26,647 subjects. Comparisons were made with Chi^2^ and Bonferroni adjustment, Kruskal–Wallis’ H with Dunnett’s post-hoc, prevalence ratios, and multivariate logistic regression. COVID-19 decreased donations of men, altruistic and repetitive donors, and increased the age of donors. HIV increased with the COVID-19 pandemic, while HBV, HCV, and HTLV I-II decreased. The pandemic had an independent effect on these viral infections. These findings constitute an alert about what may be happening in the general population and show the importance of improving epidemiological surveillance and the investigation of these infections.

## 1. Introduction

Viral infections transmitted by blood transfusion and screened at blood banks include HIV, HBV, HCV, and HTLV I-II; worldwide, they present heterogeneous prevalence. In 2021, 38.4 million people with HIV were reported, 1.5 million new cases and 650,000 deaths associated with AIDS [1]. The World Health Organization (WHO) in 2022 reported 296 million people with chronic HBV, 1.5 million new infections, and 820,000 deaths by cirrhosis or hepatocellular carcinoma (most recent data from 2019) [2]. Estimates for HCV are 58 million people with chronic infection, 1.5 million new infections, and 290,000 deaths by cirrhosis and hepatocellular carcinoma associated with this virus [3]. For HTLV I-II the information is less exhaustive, with estimates of 20 million infected, prevalence of up to 7% in endemic areas of South America and association of HTLV I with adult T-cell leukemia and tropical spastic paraparesis, while HTLV II is considered a risk factor for some types of leukemia, erythrodermatitis and peripheral neurological disorders [4].

In Colombia, the reported cases of HIV up to 2021 were 134,902, with 9210 new cases and 2112 deaths associate with AIDS [5]. Robust epidemiological data are not available for HBV; in some epidemiological bulletins (week 29 of 2021) about 922 new cases were reported, but there are no national epidemiological data [6]. The National Institute of Health, in the epidemiological surveillance protocol for hepatitis B and C, indicated that they increased 57.5% and 32.4%, respectively, in 2021 compared to 2020, without specifying the number of cases [7]. Data are only available for chronic hepatitis C, with 626 cases in 2020 [8] and 962 in 2021; This is due to the fact that this event has been monitored since 2017, in compliance with Resolution 1692, which is part of the strategy of negotiation and centralized purchase of direct-acting antivirals [9]. Of the HTLV I-II there are no official data for the country.

The foregoing shows several shortcomings in the Colombian epidemiological surveillance system, such as not notifying the majority of these viral infections, and reporting data based on passive surveillance system (without case search, based on symptomatic patients that arrive to the hospitals). For this reason, the blood bank is an ideal scenario to calculate the prevalence of these infections, since it routinely screens them in the general population that donates blood components in clinics and in extramural campaigns (those carried out outside hospitals, in factories, universities and public places).

A study of blood banks in Bogotá, Colombia in 2020 reported seroprevalence of HIV 0.17%, HBV 0.08% with HBsAg and 0.70% with anti-HBcore (HBc), HCV 0.38% and HTLV I-II 0.16% [10]. In Medellín the seroprevalences were HIV 0.12%, HBV 0.15% with HBsAg and 1.45% with anti-HBc, HCV 0.42% and HTLV I-II 0.22% [11]. These studies show divergences in the magnitude of the infection in the main cities of the country, as well as in the associated factors such as age, sex, and the type of donor (first time, repetitive, altruistic, replacement, etc.). The combined measurement of all the blood banks from Colombia reported 1.23% anti-HBc, 0.32% HCV, 0.19% HIV, 0.17% HTLV-I-II and 0.10% HBsAg [10].

It is likely that this epidemiological profile has changed due to the COVID-19 pandemic, which collapsed the health system and changed lifestyles. Particularly for blood banks, this pandemic generated shortages of blood components, forcing staff to change their donation promotion actions and the dynamics of accepting blood donors to protect patients from COVID-19. As it was a new disease, its infectious capacity by transfusion was unknown, so other reasons for donors’ rejection such as respiratory diseases, COVID-19 vaccine or contact with positive patients were included in the selection protocol [12,13]. However, in Colombia the impact of the COVID-19 pandemic on the seroprevalence of these infections, and their interaction with other risk factors has not been evaluated. It has only been indicated that the COVID-19 pandemic reduced the number of donors by14.3% during the first year. Currently, the deferrals due to COVID-19 are one week from the application of the vaccine (if it did not present adverse effects) for vaccines approved by the Ministry of Health [14,15].

In synthesis, Colombia does not have a good epidemiological surveillance system for HIV, HBV, HCV and HTLV I-II, the most rigorous data to know the prevalence of these infections in the general population are found in blood banks, these health institutions changed their dynamics of recruitment of donors due to the COVID-19 pandemic, no research has been carried out in the country on the impact of COVID-19 on the epidemiological profile of transfusion-transmissible viral infections, and the possible interaction of the COVID-19 pandemic with other risk factors for these viruses is unknown.

Therefore, the objective of this research was to analyze the impact of the COVID-19 pandemic on seroprevalence of HIV, HBV, HCV and HTLV I-II in donors from a blood bank in Medellin, Colombia, 2019–2022, and analyze the simultaneous effect of the pandemic with other risk factors.

## 2. Materials and Methods

### 2.1. Type of Study

Cross-sectional analytical.

### 2.2. Population

We included 50,561 donors who attended the blood bank of the School of Microbiology, Universidad de Antioquia, between January 2019 and December 2022; they met the criteria of the “Guide for the selection of blood donors in Colombia” [15]. We excluded donors with more than 20% of study variable data missing, donations different from whole blood and apheresis (erythropheresis and plateletpheresis), for example, stem cell donors, and records with illogical information (for example, ages under 18 or over 65).

### 2.3. Study Groups

(i) pre-pandemic from January 2019 to 19 March 2020, with 14,879 donors;
(ii) preventive isolation that began on 20 March 2020 (although officially decreed on the 25th), would end on 30 August, but due to WHO guidelines and the country’s epidemiological data, it was extended until 30 November 2020. This period includes a final phase called flexible isolation where mitigation and openings were made for some sectors and socioeconomic activities, but that did not normalize the dynamics of donor recruitment in blood banks; this group included 9035 donors;
(ii) isolation selective that occurred when reaching the plateau with a tendency to reduce cases; only people with cough, fever, respiratory distress or confirmed diagnosis were isolated, as well as people with close contact to confirmed cases. This stage was followed by the final phase called the new normality, but the donor recruitment process in these two phases was similar, for this reason they were grouped into the same category with 26,647 donors. This group includes donors recruited over two years (2021 and 2022) in order to have updated data and because the dynamics of donor recruitment in the clinics and in extramural campaigns has not returned to pre-pandemic normality, given that people and factories had changed their perceptions and behaviors regarding blood donation and in some cases do not allow access to bank staff.

### 2.4. Description of Detection Tests

For HIV, a fourth-generation qualitative immunoassay was used with simultaneous detection of the p24 antigen of HIV-1 and antibodies to HIV-1 and HIV-2 in serum, plasma or whole blood; it can distinguish acute infection from established infection. It has a sensitivity of 99.9%, and the specificity is 100% in individuals with low risk of infection (although regional validation data of the assays are not available) [16].

In HBV the detection tests were HBsAg and antibodies against the core antigen (anti-HBc) IgM/IgG class. HBsAg is the first serological marker of infection, it appears 1–10 weeks post-exposure and 2–8 weeks before of clinical symptoms, indicating ongoing or old infection. An immunoassay in serum or plasma was used with a sensitivity of 99.8% and a specificity of 100% (regional validation data are not available) [17]. Anti-HBc detects acute infection [17] and appears in the serum of those infected 1–4 weeks after the appearance of HBsAg, at the onset of symptoms, and generally remains detectable for the rest of life, indicating current or previous infection; the sensitivity is 99.5% and the specificity is 99.9% (regional validation data are not available) [18].

For HCV, IgG/IgM conjugated against recombinant antigens of the core, NS3 and NS4 regions of the HCV genome were detected, with 100% sensitivity and 99.9% specificity (regional validation data are not available) [19]. For HTLV I-II, a chemiluminescent-based test was used to detect antibodies against HTLV I-II with 100% sensitivity and 99.7% specificity (regional validation data are not available) [20].

### 2.5. Data Collection

Secondary source of information was used, contained in the Hexabank software, license 1.28.30.50. The results of the tests of the infectious agents were extracted, and the independent variables were the date of donation, age, sex, place of recruitment (in the clinic or extramural campaign), frequency of donation classified as first-time, non-repetitive (with several donations in the bank, but in periods longer than one year) and repetitive (at least two donations in a year in the blood bank), and type of donor (altruistic or replacement). This information was provided by the donors at the beginning of the donation process.

### 2.6. Control of Biases

To control selection biases, all the records of the donors were included, for whom the self-exclusion survey was applied, verification of compliance with the requirements to donate and completeness of the records was conducted. In the control of information biases, quality control of the data from the primary source was carried out, the personnel who entered the data were trained for this task, and random corroboration of 10% of the information registered in the database was carried out.

For the dependent variables, the blood bank used tests with excellent diagnostic validity and performance. Also strict quality control was conducted with the following activities: (i) dependent internal quality control, with the verification of the validity of the calibration of each serological marker; (ii) assembly of repetitions of the tube and of the unit of blood for reactive results or in the area of uncertainty; (iii) external quality control with the ProgBA program in Argentina with certification according to ISO 9001:2008 and with accreditation scope in serology according to ISO/IEC 17043:2010; (iv) external quality control of the National Institute of Health; and (v) participation in reference and counter-reference with the departmental laboratory of Antioquia (concordance 100%).

To control information biases in the secondary source, intraobserver and interobserver reproducibility of the extraction of the variables was evaluated; the first, collecting the information at two different moments and the second with the comparison of the information completed by two researchers. We used Kappa coefficient for the qualitative variables and the intraclass correlation coefficient for the age.

### 2.7. Statistical Analysis

The variables were described with frequencies (n-%) and age with median and interquartile range due to their non-normal distribution, according to the Kolmogorov-Smirnov test. The comparison of the sociodemographic and donation-related variables with the seroprevalence of the infections in the three study groups was carried out with Pearson’s Chi-square and Bonferroni adjustment. The strength of the association between the infection and the study group was determined with prevalence ratios using the Katz method, and their 95% confidence intervals, taking the pre-pandemic group as the reference group. Age and study group were compared using the Kruskal–Wallis H test and Dunnett’s post-hoc test.

Confounding variables were identified; that is, they were associated with the seroprevalence of infection, with another sociodemographic or donation-related variable, and with the study group. With these variables, a logistic regression model was performed with two purposes: excluding confounders and identifying the variables associated with the seroprevalence of transfusion-transmissible viral infections. In the logistic regression models, dummy models were constructed defining the pre-pandemic group as the reference group, and the group with the lowest seroprevalence in the other variables. Goodness-of-fit was assessed using the Wald and Hosmer-Lemeshow statistic.

The presence of interaction was ruled out using logistic multivariate models, in which the independent variables were introduced (for example, variables A, B, C and D) with the interaction factors for each one (for example, A^x^B, A^x^C, A^x^D), with the following formulation: Y = β0 + β1X1 + β2X2 + β3X3 + βnXn + β1(X1^x^X2) + β1(X1^x^X3) + β1(X1^x^Xn). Analyzes were performed in SPSS 27.0 with 95% confidence.

### 2.8. Ethics

The study was approved by the scientific committee of the blood bank, applying the guidelines of resolution 8430 of the Colombian Ministry of Health, according to which it was a risk-free study. Resolution 1995 of 1999 was also applied, which establishes standards for the management of medical charts, and in which the health team can access this information. National Decree 1377 of 2013 partially regulates Law 1581 of 2012 about protection of personal data. WHO recommendations to fill out informed consent and guarantee confidentiality, advice, quality of results and referral to health services for treatment were applied. The information in the database was managed with a code to guarantee the confidentiality of the information (unlinked information).

## 3. Results

Based on the reports of the Colombian Ministry of Health about the periods in which the COVID-19 pandemic developed in Colombia, the three groups compared were the following: (i) pre-pandemic (1 January 2019–19 March 2020); (ii) preventive isolation that included a final phase called flexible isolation (20 March 2020–30 November 2020); and (iii) isolation selective and the final phase called the new normality (1 December 2020–31 December 2022). All the characteristics in Table 1 present statistical differences in the three study groups, except the age, which was only different in the pre-pandemic group, and the type of extraction, which was different in group 3 (selective isolation and new normality). In general, the COVID-19 pandemic had an impact on the donation profile, decreasing donations of men, altruistic and repetitive donors, and those made during campaigns or outside the clinic, while the median age of donors increased. Despite the fact that in the third group some of these values increased, the figures for the pre-pandemic group have not yet been achieved (Table 1).

The seroprevalence of HIV, HBV and HTLV I-II presented statistical differences in the three study groups (Table 2). HIV increased with the COVID-19 pandemic, being 89% higher in the group of selective isolation and new normality, compared to the pre-pandemic group. HBV, according to HBsAg, decreased 53% during preventive isolation compared to pre-pandemic; for the group of selective isolation and new normality its prevalence increased until it was statistically equal to the pre-pandemic group. Anti-HBc decreased 34% during preventive isolation and 21% during selective and new normality, compared with pre-pandemic. HTLV I-II decreased 42% for the group of selective isolation with new normality (Table 3).

Multivariate logistic regression demonstrated the impact of the COVID-19 pandemic, independent of other risk factors. The pandemic increased the seroprevalence of HIV, to this was added the frequency of donation, with HIV infection being three times higher in non-repetitive and first-time donors, compared to repetitive donors. For anti-HBc the associated factors were the COVID-19 pandemic (which decreased its seroprevalence), sex (52% higher in men), the type of donation (46% higher in replacements) and the frequency of donation (two and three times higher in non-repetitive and first-time, respectively). For HTLV I-II, the associated factors were the COVID-19 pandemic, sex (72% higher in women) and the frequency of donation (Table 4).

## 4. Discussion

The COVID-19 pandemic changed the profile of donors, increased the median age and decreased donations in men, altruistic or voluntary, and repetitive donors. Bermudez-Forero et al. in 83 blood banks in the country found a decrease of 18.4% in voluntary donors and an increase of 37% in replacement donors [21]. This is important because it shows that the COVID-19 pandemic led to the failure to comply with WHO recommendations regarding favoring the collection of blood from voluntary donors, and the progressive elimination of the replacement donor, due to the greater risk of infections that are transmitted in the last group [22].

HIV increased 89% compared with pre-pandemic, this coincides with a similar study conducted at a blood bank in India that found HIV seroprevalence of 0.03% in 2019, 0.05% in 2020, and 0.09% in 2021 [23]. According to research in Canada, the public health actions aimed at reducing the transmission of SARS-CoV-2 increased the transmission of some HIV clusters [24]. A mathematical model in the Chinese population shown increases in HIV between 5% and 14% caused by the COVID-19 pandemic [25]. The factors that explain these changes include reduction in condom use during lockdown periods, and access barriers to antiretroviral therapy, pre-exposure prophylaxis, post-exposure prophylaxis, counseling, and diagnostic services [26,27]. However, more studies are needed to test this hypothesis, as well as local investigations to confirm whether what occurs in donors is similar to the general population.

The seroprevalence of HBV and HCV in donors from this blood bank decreased compared with pre-pandemic, this contrasts with a study of a blood bank in India which reported an increase of HBV and HCV [23]. In a study with health workers who treated patients with HCV or HBV, and coordinators of hepatitis programs from 44 countries, 21% (of the countries) reported a decrease of more than 50% in treatments, and 28% (of the countries) reported a decrease higher than 50% in diagnostic tests and disruptions in supply chains [28]. In Colombia a study reported that the pandemic decreased testing for HCV antibodies, HCV viral load, HCV genotyping, HBsAg, HBV antibodies, liver function tests, follow-up visits, and follow-up by hepatology specialties [8]. These interruptions in supply chains, diagnostic services, and treatment for hepatitis during the COVID-19 pandemic, led to an increase in viral hepatitis in the short term and could become a source of long-term transmission [29].

Some authors have explained these findings with changes in sexual behavior as a result of COVID-19 and periods of confinement, which could have interrupted the transmission of the virus. A meta-analysis showed a significant decrease in the number of sexual interactions during the COVID-19 pandemic; up to 60% of participants without sexual relationships, and a decrease in the frequency of sexual activity up to 53% [30]. This evidence would suggest a similar behavior for HIV, but this trend did not occur in this population, so the hypothesis of underreporting of HBV would be stronger, due to the previously mentioned factors, and by taking into account that the data in the selective isolation and new normality group tend to equal the pre-pandemic cases. On the other hand, the decreasing trend in HCV could be explained, in the Colombian case, by the explicit efforts in Resolution 1692 of 2017 or the strategy for negotiation and centralized purchase of direct-acting antivirals for chronic hepatitis C [9].

Regarding HTLV I-II, there was a decrease seroprevalence up to 0.15%. It is not possible to compare this finding with studies in other contexts due to the lack of data; however, a meta-analysis on the impact of disasters on the safety of donated blood (proxy of atypical emergencies such as the COVID-19 pandemic), did not detect statistical differences in HTLV I-II; however, the quality of the evidence synthesized in the meta-analysis was deficient [31]. On the other hand, an investigation carried out in the same blood bank form Medellín, Colombia, in the period 2014–2018, found a seroprevalence of HTLV I-II of 0.176% (95% CI = 0.139–0.213) [32], so that the results of this study in the group of absolute preventive isolation and selective isolation and new normality are within the confidence intervals of the previous investigation; the atypical year was precisely 2019 when HTLV I-II increased up to 0.26%. This result shows that the factors that affect the seroprevalence of this virus in blood donors are complex, and more research is needed in this topic.

In addition to the impact of the COVID-19 pandemic, it should be noted that the main factors associated with the seroprevalence of the viruses were the type of donation (higher in replacement/family donors) and the type of donor (higher in non-repetitive and first-time donors). Studies that have compared the prevalence of transfusion-transmissible infections between altruistic and replacement donors, and have found higher prevalence among family/replacement donors [33,34,35]. Several reasons explain this finding:

(i) Altruistic donors donate their blood by free will and without pressure, while family/replacement donors feel that it is necessary to donate for fear of the death of their relatives and during the selection process they tend to hide high-risk behaviors or diseases [35].
(ii) Due to the prohibition of buying blood officially, the responsibility of bringing donors to the blood bank has been transferred to the patients, her friends or relatives. This can result in people who come forward to donate having been financially recruited by the patient or their family. Particularly people from rural areas who go to hospitals in large cities, find it difficult to obtain donors from the required group and may fall prey to unofficial blood sellers [35].
(iii) Voluntary donors have better lifestyles and a higher level of education. Although educational status is not always protective against sexually transmitted infections (STIs) [36], it has been described that education is an independent determinant of STIs among clients consulting Dutch sexual health centers [37] and that education protects against behaviors that are harmful to health and raises awareness among blood donors about the risks of transmitting viral infections through their donations [33].
(iv) Altruistic donors are younger, so they had better access to vaccines in their childhood [38].

This highlights the importance of the aforementioned WHO recommendation to favor the collection of blood from voluntary or altruistic donors, and progressively eliminate replacement donations [22].

### 4.1. Limitations

This study has the following limitations: the associations found in the bivariate and multivariate analyzes are exploratory; they are sources of hypotheses and do not indicate causality. The world literature in this field of knowledge is scarce and little is known about the sexual behavior of Colombians during the pandemic, which prevented us from exploring other explanations for our findings. The COVID-19 pandemic was faced with different coercive measures depending on the country or even within regions of the same country, a situation that can affect social interaction and therefore the circulation of a virus, so the comparisons by year can be affected.

### 4.2. Strengths

This is one of the first studies in the world that evaluates the impact of the COVID-19 pandemic on the seroprevalence of the HIV, HBV, HCV and HTLV I-II in blood donors. It presents seroprevalence in a healthy population with a high sample size, which gives more precision to the inferences, and improves the understanding of the epidemiological situation of these infections in Colombia, a country in which the magnitude and risk factors in the general population are unknown, given that surveillance is mainly done with clinical cases or captured in health institutions.

## 5. Conclusions

The COVID-19 pandemic changed the profile of donors in the blood bank (increasing replacement donors), it increased HIV and decreased diagnostic efforts for HBV. These findings constitute an alert about what may be happening in the general population and show the importance of improving surveillance and epidemiological investigation of these infections in Colombia.

## Figures and Tables

**Table 1:** Comparison of sociodemographic and donation characteristics in the three study groups.

	Pre-Pandemic % (*n*)	Absolute Preventive Isolation % (n)	Selective Isolation and New Normality %(n)	p Chi^2^
**Sex**				
Women	54.0 (8034) ^b^	59.9 (5415) ^a^	56.8 (15,137)	<0.001
Men	46.0 (6845) ^a^	40.1 (3620) ^b^	43.2 (11,510)	
**Donor type**				
Altruistic	83.6 (12,445) ^a^	79.7 (7202) ^b^	81.5 (21,705)	<0.001
Replacement	16.4 (2434) ^b^	20.3 (1833) ^a^	18.5 (4942)	
**Donation frequency**				
Not repetitive	39.6 (5890) ^b^	47.0 (4243)	45.8 (12,215)	<0.001
First time	36.1 (5377) ^a^	33.0 (2982)	29.7 (7919) ^b^	
Repetitive	24.3 (3612)	20.0 (1810) ^b^	24.4 (6513)	
**Collection**				
Clinic	22.8 (3390) ^b^	26.8 (2420) ^a^	24.6 (6567)	<0.001
Campaign	77.2 (11,489) ^a^	73.2 (6615) ^b^	75.4 (20,080)	
**Extraction**				
Whole blood	92.3 (13,733)	92.8 (8382)	94.1 (25,070) ^a^	<0.001
Aphaeresis	7.7 (1146)	7.2 (653)	5.9 (1577) ^b^	
**Age**				**p KW ^c^**
Median (IR) ^c^	31 (23–43) ^a^	33 (25–45)	34 (25–45)	<0.001

**^a^** Statistically higher subgroup (compared to its row values) according to post-hoc. **^b^** Statistically lower subgroup (of its row). **^c^** KW: Kruskal–Wallis. IR: interquartile range.

**Table 2:** Seroprevalence of viral infections detected in the blood bank in the three study groups.

	Pre-Pandemic % (n)	Absolute Preventive Isolation % (n)	Selective Isolation and New Normality %(n)	p Chi^2^
HIV	0.09 (14)	0.13 (12)	0.17 (46)	<0.05
HBV HBsAg	0.19 (28)	0.09 (8)	0.16 (43)	<0.05
HBV anti-HBc	1.30 (193) ^a^	0.86 (78)	1.03 (276)	<0.05
HTLV I-II	0.26 (38)	0.17 (15)	0.15 (40)	<0.05
HCV	0.49 (73)	0.44 (40)	0.39 (103)	0.287

**^a^** Statistically higher subgroup (compared to its row values) according to post-hoc.

**Table 3:** Prevalence ratios of viral infections with differences in study groups.

	Groups with Statistical Differences	Prevalence Ratio (CI95%)
HIV	Selective isolation and new normality/Pre-pandemic	1.89 (1.01–3.33) *
HBV HBsAg	Absolute preventive isolation/Pre-pandemic	0.47 (0.21–0.99) *
HBV anti-HBc	Absolute preventive isolation/Pre-pandemic	0.66 (0.51–0.86) **
	Selective isolation and new normality/Pre-pandemic	0.79 (0.66–0.95) *
HTLV I-II	Selective isolation and new normality/Pre-pandemic	0.58 (0.38–0.92) *

CI—confidence interval. * *p* < 0.05; ** *p* < 0.01.

**Table 4:** Multivariate regression for factors associated with HIV, HBV (anti-HBc) and HTLV I-II seroprevalence.

Model for Each Infection	B	Error	Wald	Prevalence Ratio (CI95%)
**HIV**				
*Study group*				
Absolute preventive isolation/Pre-pandemic	0.32	0.39	0.64	1.42 (0.66–3.08)
Selective isolation and new normality/Pre-pandemic	0.62	0.31	4.01	1.91 (1.05–3.48) *
*Donation frequency*				
Non-repetitive/Repetitive	1.18	0.44	7.21	3.43 (1.44–8.14) **
First time/Repetitive	1.29	0.45	8.29	3.26 (1.34–7.91) **
**HBV anti-HBc**				
*Study group*				
Absolute preventive isolation/Prepandemic	−0.52	0.14	14.71	0.59 (0.46–0.78) **
Selective isolation and new normality/Pre-pandemic	−0.28	0.10	8.99	0.75 (0.62–0.91) **
*Sex*				
Men/Women	0.42	0.09	22.89	1.52 (1.28–1.80) **
*Donation type*				
Replacement/Altruistic	0.38	0.10	14.94	1.46 (1.20–1.76) **
*Donation frequency*			70.15	
Non-repetitive/Repetitive	0.75	0.14	28.93	2.12 (1.61–2.79) **
First time/Repetitive	1.20	0.15	67.81	3.33 (2.50–4.43) **
*Age*	0.05	0.00	190.40	Not interpretable
**HTLV I-II**				
*Study group*				
Absolute preventive isolation/Pre-pandemic	−0.47	0.31	2.41	0.62 (0.34–1.13)
Selective isolation and new normality/Pre-pandemic	−0.53	0.23	5.35	0.59 (0.38–0.92) *
*Sex*				
Women/Men	0.54	0.23	5.72	1.72 (1.10–2.69) *
*Donation frequency*			7.91	
Non-repetitive/Repetitive	0.83	0.35	5.53	2.29 (1.15–4.55) *
First time/Repetitive	1.00	0.35	7.89	2.71 (1.35–5.43) **

CI—confidence interval. * *p* < 0.05; ** *p* < 0.01. No multivariate associations were found for HBV_HBsAg, only the association with the study group was statistically significant.

## Data Availability

All relevant data are cited in the manuscript.

## References

[1] ONUSIDA Últimas Estadísticas Sobre el Estado de la Epidemia de Sida. https://www.unaids.org/es/resources/fact-sheet#:~:text=38%2C4%20millones%20%5B33%2C,con%20el%20sida%20en%202021.

[2] World Health Organization Hepatitis B. https://www.who.int/es/news-room/fact-sheets/detail/hepatitis-b#:~:text=La%20OMS%20estima%20que%20296,(c%C3%A1ncer%20primario%20del%20h%C3%ADgado).

[3] World Health Organization Hepatitis C. https://www.who.int/es/news-room/fact-sheets/detail/hepatitis-c.

[4] Organización Panamericana de la Salud Términos de Referencia para la Contratación de un/a Profesional para Apoyar la Revisión Sistemática de Evidencia y Construcción del Lineamiento de Atención Clínica a las Personas con HTLV 1 y 2 en Colombia. https://www.paho.org/sites/default/files/022_21_tdr_lineamiento_htlv_1_y_2.pdf.

[5] Cuenta de Alto Costo (2021). Fondo Colombiano de enfermedades de alto costo. Situación del VIH y SIDA en Colombia. https://cuentadealtocosto.org/site/wp-content/plugins/pdfjs-viewershortcode/pdfjs/web/viewer.php?file=https%3A%2F%2Fcuentadealtocosto.org%2Fsite%2Fwp-content%2Fuploads%2F2022%2F02%2FCAC.Co_Libro_Sit_VIH2021_v8.pdf&download=false&print=true&openfile=false.

[6] Instituto Nacional de Salud Boletín Epidemiológico Semanal. Comportamiento de la Notificación de de Hepatitis B., C y B-D. http://www.saludcapital.gov.co/CTDLab/Publicaciones/2021/Boletin_epidemiologico-semana_29-2021.pdf.

[7] Instituto Nacional de Salud Protocolo de Vigilancia de Hepatitis B, C y coinfección/superinfección Hepatitis B-Delta. https://www.ins.gov.co/buscador-eventos/Lineamientos/Pro_Hepatitis%20BCD.pdf.

[8] Cuenta de Alto Costo (2020). Fondo Colombiano de Enfermedades de Alto Costo. Situación de la Hepatitis C Crónica en los Regímenes Subsidiado y Contributivo de Colombia. https://cuentadealtocosto.org/site/wp-content/uploads/2021/07/CAC.Co_2021_07_14_Libro_Sit_Hepatitis%20C_2020_v3(1).pdf.

[9] Cuenta de Alto Costo Fondo Colombiano de Enfermedades de Alto Costo. Día Mundial de la Hepatitis 2022. https://cuentadealtocosto.org/site/hepatitis-c/dia-mundial-de-la-hepatitis-2022/.

[10] Red Distrital de Bancos de Sangre, Servicios de Transfusión Sanguínea y Terapia Celular (2020). Boletín Annual. http://www.saludcapital.gov.co/DDS/Boletin%20Estadistico/Boletin_Estadistico_Red_Sangre_2020.pdf.

[11] Flórez-Duque J., Cardona-Arias J. (2016). Marcadores de Infecciones Transmisibles vía Transfusional: El Caso del Banco de Sangre de la Escuela de Microbiología de la Universidad de Antioquia, 2015–2016.

[12] Routray S.S., Ray G.K., Prakash S., Sahu A., Naik A., Mukherjee S. (2022). Impact of COVID-19 on blood donor deferral patterns during the COVID-19 pandemic: A retrospective analysis. Vox Sang..

[13] Ngo A., Masel D., Cahill C., Blumberg N., Refaai M.A. (2020). Blood Banking and Transfusion Medicine Challenges During the COVID-19 Pandemic. Clin. Lab. Med..

[14] Gupta A.M., Jain P. (2021). Blood donor deferral periods after COVID-19 vaccination. Transfus. Apher. Sci..

[15] Instituto Nacional de Salud, República de Colombia (2022). Lineamiento Técnico para la Selección de Donantes de Sangre en Colombia (actualización agosto de 2022). https://www.ins.gov.co/bibliotecadigital/seleccion-donantes-sangre.pdf.

[16] Abbott laboratories (2014). HIV-1/2 Ag/Ab Combo. https://www.globalpointofcare.abbott/en/product-details/determine-1-2-ag-ab-combo.html.

[17] Instituto Nacional de Salud, República de Colombia (2010). Respecto a la Confirmación, Asesoría, Canalización a los Servicios de Salud y Reporte al Sistema de Vigilancia Epidemiológica, de Donantes de Sangre con Pruebas Tamiz Doblemente Reactivas para Marcadores Infecciosos en Bancos de Sangre de Colombia.

[18] Abbott Laboratories (2014). Hepatitis B Virus Core Antigen (E. coli, Recombinant).

[19] Abbott Laboratories (2019). Anti HCV Hepatitis C Virus Encoded Antigens (Recombinant c100-3, HCr43). https://www.fda.gov/media/128755/download.

[20] Abbott Laboratories (2014). HTLV-I/HTLV-II.

[21] Bermúdez-Forer M.I., Soto-Viáfara J.A., Gardeazábal-Acuña P.A., Anzola-Samudio D.A., García-Otálora M.A. (2021). Effect of the first year of COVID-19 pandemic on the collection and use of blood components in Colombia monitored through the national haemovigilance system. Transfus. Med..

[22] World Health Organization (2012). Blood Donor Selection: Guidelines on Assessing Donor Suitability for Blood Donation.

[23] Kaur P., Bedi R.K., Mittal K., Sood T. (2022). Exploring the unseen effect of COVID 19 pandemic on blood transfusion services in a tertiary care centre. Transfus. Apher. Sci..

[24] Miller R.L., McLaughlin A., Montoya V., Toy J., Stone S., Harding J., Liang R.H., Wong J., Barrios R., Montaner J.S.G. (2022). Impact of SARS-CoV-2 lockdown on expansion of HIV transmission clusters among key populations: A retrospective phylogenetic analysis. Lancet Reg. Health Am..

[25] Booton R.D., Fu G., MacGregor L., Li J., Ong J.J., Tucker J.D., Turner K.M., Tang W., Vickerman P., Mitchell K.M. (2021). The impact of disruptions due to COVID-19 on HIV transmission and control among men who have sex with men in China. J. Int. AIDS Soc..

[26] Mirzaei H., Moradi Y., Abbaszadeh S., Nasiri N., Mehmandoost S., Khezri M., Tavakoli F., Sharifi H. (2022). The Impact of COVID-19 on Disruptions of HIV-related Services: A Rapid Review. Med. J. Islam Repub. Iran.

[27] Mitchell K.M., Dimitrov D., Silhol R., Geidelberg L., Moore M., Liu A., Beyrer C., Mayer K.H., Baral S., Boily M.C. (2021). The potential effect of COVID-19-related disruptions on HIV incidence and HIV-related mortality among men who have sex with men in the USA: A modelling study. Lancet HIV.

[28] Laury J., Hiebert L., Ward J.W. (2021). Impact of COVID-19 Response on Hepatitis Prevention Care and Treatment: Results from Global Survey of Providers and Program Managers. Clin. Liver Dis..

[29] Pley C.M., McNaughton A.L., Matthews P.C., Lourenço J. (2021). The global impact of the COVID-19 pandemic on the prevention, diagnosis and treatment of hepatitis B virus (HBV) infection. BMJ Glob Health.

[30] Mourikis I., Kokka I., Koumantarou-Malisiova E., Kontoangelos K., Konstantakopoulos G., Papageorgiou C. (2022). Exploring the adult sexual wellbeing and behavior during the COVID-19 pandemic. A systematic review and meta-analysis. Front. Psych..

[31] Laermans J., Dorien O., Van den Bosch E., De Buck E., Compernolle V., Shinar E., Vandekerckhove P. (2022). Impact of disasters on blood donation rates and blood safety: A systematic review and meta-analysis. Vox Sang..

[32] Cardona-Arias J.A., Vélez-Quintero C., Calle-González O.V., Florez-Duque J., Zapata J.C. (2019). Seroprevalence of human T-lymphotropic virus HTLV and its associated factors in donors of a blood bank of Medellín-Colombia, 2014–2018. PLoS ONE.

[33] Abdel Messih I.Y., Ismail M.A., Saad A.A., Azer M.R. (2014). The degree of safety of family replacement donors versus voluntary non-remunerated donors in an Egyptian population: A comparative study. Blood Transfus..

[34] Zhou Q., Liu A., Wang S., Li J., He M., Chen L. (2022). Hepatitis C virus screening reactive among blood donors in mainland China: A systematic review and meta-analysis. Transfus. Med..

[35] Jain R., Gupta G. (2012). Family/friend donors are not true voluntary donors. Asian J. Transfus. Sci..

[36] Annang L., Walsemann K.M., Maitra D., Kerr J.C. (2010). Does education matter? Examining racial differences in the association between education and STI diagnosis among black and white young adult females in the U.S. Public Health Rep..

[37] Slurink I.A., Götz H.M., van Aar F., van Benthem B.H. (2021). Educational level and risk of sexually transmitted infections among clients of Dutch sexual health centres. Int. J. STD. AIDS..

[38] Ropero Álvarez A.M., Pérez-Vilar S., Pacis-Tirso C., Contreras M., El Omeiri N., Ruiz-Matus C., Velandia-González M. (2017). Progress in vaccination towards hepatitis B control and elimination in the Region of the Americas. BMC Public Health.

